# CuInS_2_ quantum dot-sensitized TiO_2_ nanorod array photoelectrodes: synthesis and performance optimization

**DOI:** 10.1186/1556-276X-7-652

**Published:** 2012-11-27

**Authors:** Zhengji Zhou, Shengjie Yuan, Junqi Fan, Zeliang Hou, Wenhui Zhou, Zuliang Du, Sixin Wu

**Affiliations:** 1 Key Lab for Special Functional Materials of Ministry of Education, Henan University, Kaifeng, 475004, China

**Keywords:** CuInS_2_, Quantum dots, TiO_2_, Nanorod arrays, Photoelectrochemical properties

## Abstract

CuInS_2_ quantum dots (QDs) were deposited onto TiO_2_ nanorod arrays for different cycles by using successive ionic layer adsorption and reaction (SILAR) method. The effect of SILAR cycles on the light absorption and photoelectrochemical properties of the sensitized photoelectrodes was studied. With optimization of CuInS_2_ SILAR cycles and introduction of In_2_S_3_ buffer layer, quantum dot-sensitized solar cells assembled with 3-μm thick TiO_2_ nanorod film exhibited a short-circuit current density (*I*_sc_) of 4.51 mA cm^−2^, an open-circuit voltage (*V*_oc_) of 0.56 V, a fill factor (FF) of 0.41, and a power conversion efficiency (*η*) of 1.06%, respectively. This study indicates that SILAR process is a very promising strategy for preparing directly anchored semiconductor QDs on TiO_2_ nanorod surface in a straightforward but controllable way without any complicated fabrication procedures and introduction of a linker molecule.

## Background

Since the introduction of an important advancement of using a nanostructured dye-sensitized photo-active electrode in a solar cell by O'Regan and Grätzel in 1991 [1], the dye-sensitized solar cells (DSSCs) have attracted a lot of attention in the past two decades and been considered as a potential low-cost alternative to conventional silica-based solar cells [2-5]. The latest energy conversion efficiency of DSSCs was reported to exceed 12% [6]. Further improvement of the efficiency of DSSCs is impeded by the design of new dyes which could absorb all photons above a threshold energy of 1.3 to 1.4 eV (roughly 940 to 890 nm) without affecting the injection efficiency and regeneration rate [7,8]. Another attractive strategy is to use semiconductor quantum dot (QD) as a substitute for organic dye [9-12]. For enhancement of the conversion efficiency, it is still necessary to select a semiconducting material with the proper band gap that absorbs strongly for photon energies above 1.3 eV. Ternary chalcopyrite CuInS_2_, which is a direct band gap semiconductor with Eg = 1.55 eV (bulk) has many favorable features including high absorption coefficient (10^5^ cm^−1^) and proper band gap well matched to the solar spectrum [13,14], as well as non-toxicity and good stability. It has been demonstrated as a promising photosensitizer successfully used in quantum dot-sensitized solar cells (QDSSCs) [15,16].

Up to now, the reports on CuInS_2_-based QDSSCs are almost exploited a presynthesis method, in which the CuInS_2_ colloidal QDs are presynthesized and anchored to the electrodes by means of bifunctional linker molecules or direct adsorption [16,17]. This process suffers from rather low QD loading and relatively weaker electronic coupling between QDs and TiO_2_[18]. Another approach for QD sensitization is direct growth of QDs on TiO_2_ by successive ionic layer adsorption and reaction (SILAR), in which the ions in the precursor solution are adsorbed directly onto the bare surface of TiO_2_ to form a very thin conformal covering film [19,20]. The SILAR process has recently emerged as the best method for adsorbing QDs onto TiO_2_ electrodes, owing to its facile and reproducible preparation, high QD loading together with well controllable in size and density of the target semiconductor QDs, and efficient electron transfer to TiO_2_[18,20]. Very recently, Chang et al. have reported CuInS_2_ QD-sensitized TiO_2_ nanoparticle film by SILAR process [21]. For assembly of QDSSCs, one dimensional (1D) TiO_2_ nanostructure arrays possess the superiority over other nanomaterials due to its more open structure which was preferable for both sensitizer and electrolyte filling [22]. Moreover, 1D nanostructure can provide a direct and efficient pathway for electrons from sensitizer to conductive substrate compared to the disordered electron pathway in nanoparticles [23-26]. Therefore, TiO_2_ has been fabricated into various 1D nanostructure arrays such as nanowires (NWs), nanorods (NRs), and nanotubes for photovoltaic devices. Single-crystalline TiO_2_ NW or NR array is preferable over polycrystalline one in electron transfer because of electron scattering or trapping at grain boundaries of polycrystal [22]. However, the exploitation of CuInS_2_ QD-sensitized single-crystalline TiO_2_ NRs for QDSSCs has not been systematically investigated.

In this study, using the SILAR procedures, CuInS_2_ QDs were successfully assembled onto vertically oriented single-crystalline TiO_2_ nanorod array (NRA), which was grown directly onto transparent conductive fluorine-doped tin oxide (FTO) substrates. A detailed structural characterization and photoelectrochemical investigation of the CuInS_2_-sensitized TiO_2_ nanorod array photoelectrodes were discussed in this article. Furthermore, by introduction of a cadmium-free In_2_S_3_ buffer layer to adjust the interfacial properties of CuInS_2_ and TiO_2_, the photoelectrical properties of QDSSCs were remarkably improved.

## Methods

### Materials

Copper (II) sulfate (CuSO_4_, 99%), indium (III) sulfate (In_2_(SO_4_)_3_, 98.0%), indium(III) nitrate (99.9%), sodium sulfide (Na_2_S, 98%), and titanium butoxide (97%) were purchased from Sigma-Aldrich (Shanghai) Trading Co., Ltd. Potassium phosphate monobasic (KH_2_PO_4_, 99.99%), sodium hydroxide (NaOH, 98%), sodium sulfite (Na_2_SO_3_, 97%), and concentrated hydrochloric acid (HCl, 37% by weight) were obtained from Tianjin Chemical Reagents Company (Tianjin, China). All the materials were used directly without further purification. Triply deionized water (resistivity of 18.2 MΩ cm^−1^) was obtained from a Milli-Q ultrapure water system (EMD Millipore Corporation, MA, USA). FTO-coated glass slides (F: SnO_2_, 14 Ω/square, Nippon Sheet Glass Group, Tokyo, Japan) were thoroughly washed with a mixed solution of deionized water, acetone, and 2-propanol (volume ratios of 1:1:1) under sonication for 60 min.

### Synthesis of TiO_2_ NRAs

The TiO_2_ NRAs were grown directly on transparent FTO substrates by a hydrothermal method; details of the synthesis procedure can be found in Liu and Aydil [27]. In a typical synthesis, 30 mL of concentrated HCl was added to 30 mL of deionized water with stirring. After 5 min of stirring, 1 mL of titanium butoxide was added dropwise to the solution and stirred continuously for another 5 min to obtain a clear transparent solution. The resulting solution was then transferred into a 120-mL Teflon-lined stainless-steel autoclave. Then, one piece of cleaned FTO glass was placed into the autoclave at an angle of about 45° against the wall of the Teflon lining with the conducting side facing down. Subsequently, the autoclave was sealed and placed inside an electronic oven. The hydrothermal synthesis was conducted at 150°C for 20 h, and the obtained TiO_2_ NRAs on FTO glass substrates were taken out of the cooled autoclave, rinsed extensively with distilled water, and finally dried in air.

### Fabrication of CuInS_2_ QD-sensitized TiO_2_ NRA electrodes

CuInS_2_ QDs were attached to TiO_2_ NRAs by the SILAR process, which was similar to that described by Wu et al. [28]. Briefly, the TiO_2_ nanorod array substrate was dipped sequentially in aqueous solutions of 0.1 M In_2_(SO_4_)_3_ for 60 s, and S ion precursor solution (0.075 M Na_2_S, with pH equal to 11.3 adjusted by a buffer composed of 0.1 M KH_2_PO_4_ and 0.1 M NaOH) for 240 s, following in 0.01 M CuSO_4_ aqueous solutions for 20 s, and S ion precursor solution for 240 s. Between each dip, the films were rinsed with deionized water for 30 s to remove excess precursors and dried in air before the next dipping. Such an immersion procedure is termed as one cycle for copper indium sulfide deposition, and this immersion cycle was repeated several times until the desired amount of CuInS_2_ QDs was incorporated. To increase the crystallinity and the concentration of sulfur in the SILAR-deposited CuInS_2_, the samples were annealed in furnace under sulfur ambiance (using S powder as the S source) at 500°C for 30 min after SILAR deposition.

A In_2_S_3_ buffer layer was introduced between TiO_2_ and CuInS_2_ layer also by SILAR. For In_2_S_3_ deposition from their precursor solutions, 0.1 M indium nitrate in ethanol was used as cation source, and 0.1 M sodium sulfide in 1:1 methanol and water as anion source.

### Characterization

The as-prepared CuInS_2_ QD-sensitized TiO_2_ NRA electrodes were characterized by various analytical and spectroscopic techniques. The morphology of the sample was studied by a field-emission scanning electron microscopy (FESEM, JSM-7001 F, JEOL Co., Ltd., Beijing, China). Transmission electron microscopy (TEM), and high-resolution TEM (HRTEM) investigations were carried out by a JEOL JEM-2100(UHR) microscope operating at 200 kV. The samples were detached from the FTO substrate, then dispersed in ethanol by sonication, and dropped onto a carbon film supported on a copper grid. Structure characterizations of the CuInS_2_-sensitized TiO_2_ NRA films were conducted using X-ray diffraction (XRD). The XRD patterns were recorded using a Philips X'Pert PRO X-ray diffractometer (Royal Philips Electronics, Amsterdam, The Netherlands) with Cu Kα1 radiation (*λ* = 1.5406 Å) from 20 to 70° at a scan rate of 2.4° min^−1^. X-ray tube voltage and current were set at 40 kV and 40 mA, respectively. The absorption spectra for CuInS_2_ QD-sensitized TiO_2_ NRA electrodes were recorded on a CARY5000 UV-visible NIR spectrometer (Agilent Technologies Inc., CA, USA).

### Photoelectrochemical measurements

To examine the photovoltaic properties of CuInS_2_ QD-sensitized TiO_2_ NRA electrodes, the electrodes were assembled into cells using a Pt-coated counter-electrode facing it, which had been prepared by sputtering with 100 nm of Pt on cleaned FTO glass using radio frequency sputtering at a power of 150 W and a working pressure of 3 × 10^−3^ Torr with argon gas for 60 s. The sandwich-type solar cells were then sealed with 60-μm thick hot-melt film (Surlyn 1702, Dupont, DE, USA) by hot pressing. Polysulfide electrolyte consisting of 0.24 M Na_2_S and 0.35 M Na_2_SO_3_ in aqueous solution was injected into the interelectrode space by capillary force. A mask with an aperture of 0.16 cm^2^ (0.4 cm × 0.4 cm) was used to define the active area of the cell and prevent stray light from producing photocurrents. The photocurrent-voltage (*I-V*) curves were measured under an illumination of a solar simulator (Oriel class A, SP91160A, Newport Corporation, CA, USA) at one sun (AM1.5, 100 mW cm^−2^) irradiation calibrated with a Si-based reference. A Keithley model 2400 digital source meter (Keithley Instruments, Inc., OH, USA) was used to record the *I-V* characteristics by applying an external bias potential to the cell and measuring the photocurrent.

## Results and discussion

### Characterization of CuInS_2_ QD-sensitized TiO_2_ nanorod array

**Figure 1:**
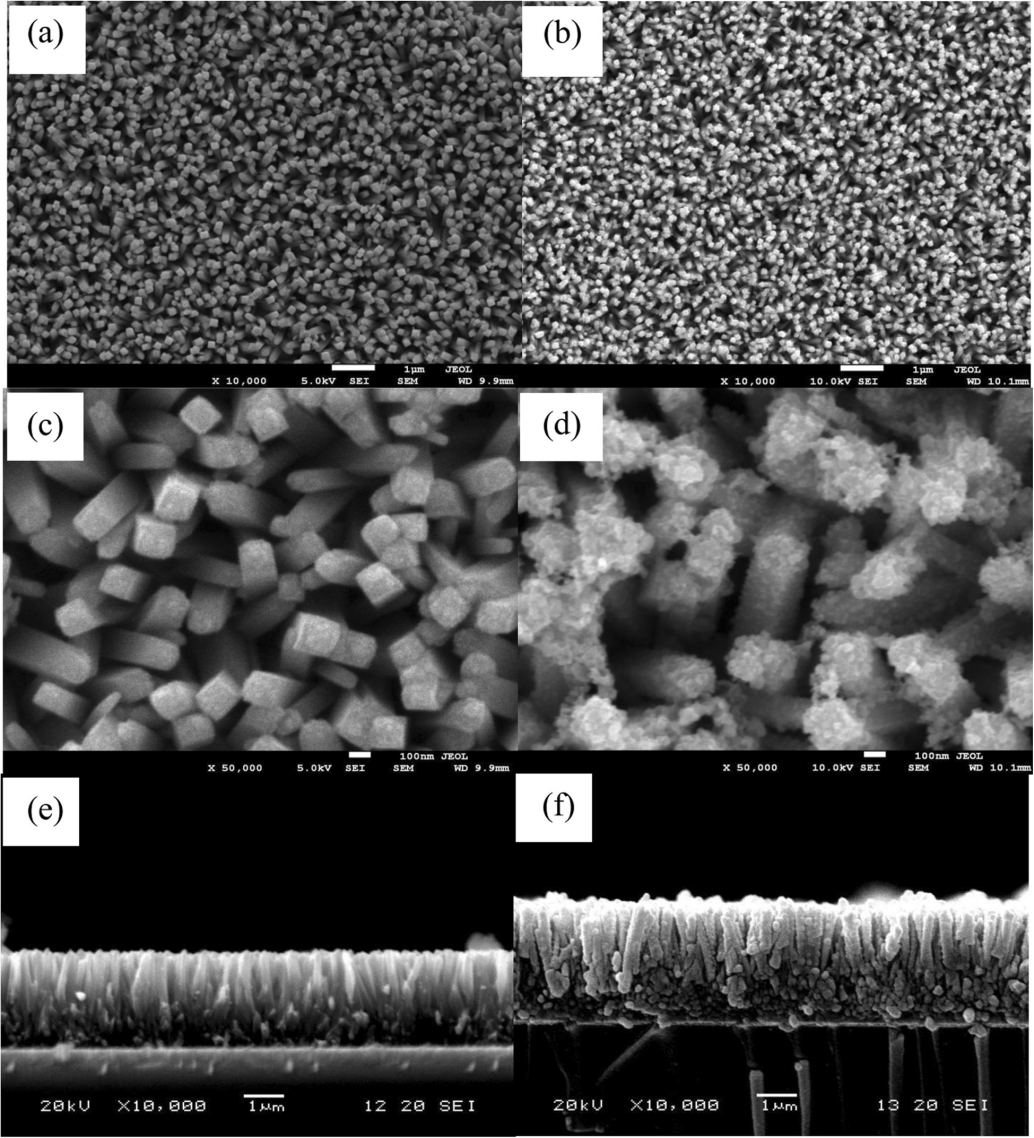
**FESEM images.** (**a**) TiO_2_ nanorod array (top view), (**b**) TiO_2_ NRAs after 7 cycles of CuInS_2_ SILAR deposition (top view), (**c**) and (**d**) are the corresponding larger magnification images of (a) and (b), (**e**) and (**f**) are the cross-sectional SEM images of TiO_2_ NRAs grown on FTO substrate before and after CuInS_2_ deposition.

Figure 1a,b shows the typical FESEM images of the bare TiO_2_ NRA films and CuInS_2_ QD-sensitized TiO_2_ NRA films. It is clear that the entire surface of the FTO substrate is covered uniformly and densely with vertical alignment of TiO_2_ nanorods. From the higher magnification (Figure 1c) and cross-sectional view (Figure 1e) of such array, the average diameter and length of nanorods are 90 nm and 3.0 μm, respectively, and the sides of the TiO_2_ nanorods are relatively smooth. The nanorods are tetragonal in shape, which is the expected growth habit for the tetragonal crystal. After assembled with CuInS_2_ QDs for 7 cycles, the vertically aligned TiO_2_ nanorod array structure is retained, as shown in Figure 1b. However, it is revealed that the average diameter of the nanorods increases, and the surface becomes rather rougher and possesses uniform particles in the enlarged FESEM image (Figure 1d). Figure 1f is a cross-sectional view of the CuInS_2_ QD-sensitized TiO_2_ NRAs, showing that the CuInS_2_ QDs have been uniformly deposited onto the surfaces of TiO_2_ nanorods along their major length.

**Figure 2:**
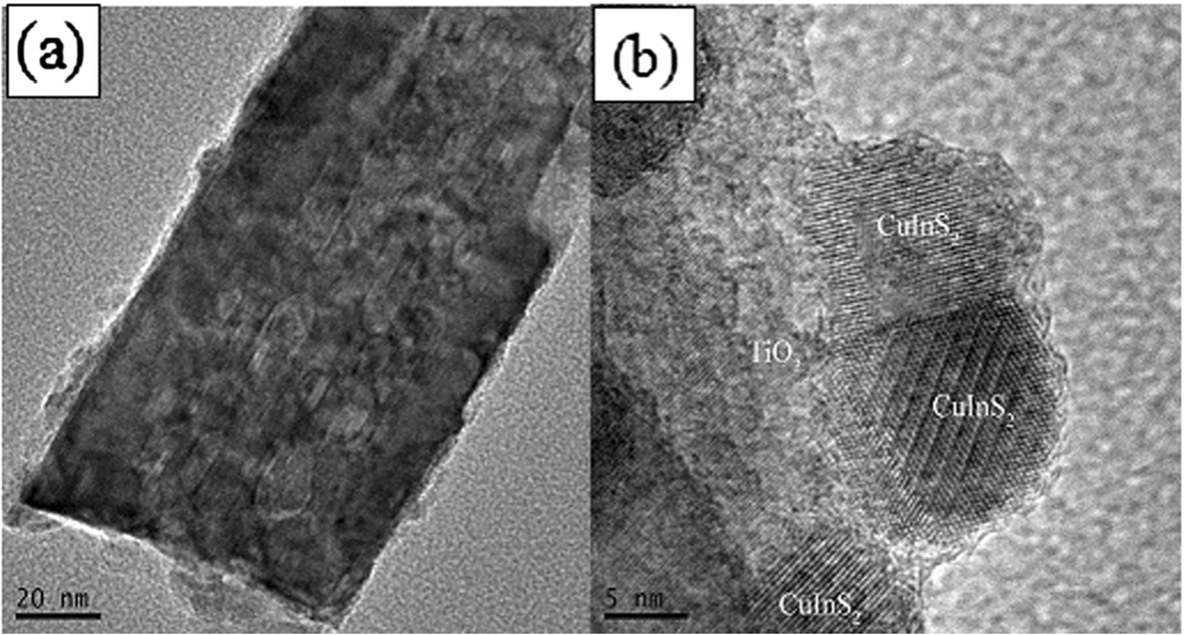
**TEM (a) and HRTEM (b) images of CuInS_**2 **_quantum dots.** The CuInS_2_ quantum dots are deposited onto a TiO_2_ nanorod; the SILAR cycle number for CuInS_2_ deposition was 7.

It is important to directly observe the QD sensitizers on TiO_2_ surface. The TEM and HRTEM can provide detailed microscopic information on the size of QDs and their distribution over the TiO_2_ nanorods, which is crucial in understanding how they are deposited and how they would affect the photoelectrochemical properties of electrodes. Figure 2a shows the TEM image of a TiO_2_ nanorod deposited with CuInS_2_ for 7 SILAR cycles, displaying that the bare surface of TiO_2_ nanorod appears to be covered by a thin shell consisting of a large amount of smaller dots. Figure 2b shows the HRTEM image at the edge side of TiO_2_ nanorod, indicating the high crystallinity of TiO_2_ and CuInS_2_. The larger crystallite appearing in the left region of the image is identified to be TiO_2_, and the observed lattice spacing of 0.322 nm corresponds to the (110) plane of tetragonal rutile TiO_2_. The randomly oriented crossed fringe patterns with *d* = 0.320 nm on the edge of the nanorod can be assigned to the (112) planes of the tetragonal CuInS_2_; they did not have preferential alignment along the rod axis, and the diameter of the single-crystalline QDs was about 5 to 10 nm. In addition, EDS analysis shows that the ratio of Cu/In/S is 1.02:1.00:1.91 (Additional file 1: Figure S1).

**Figure 3:**
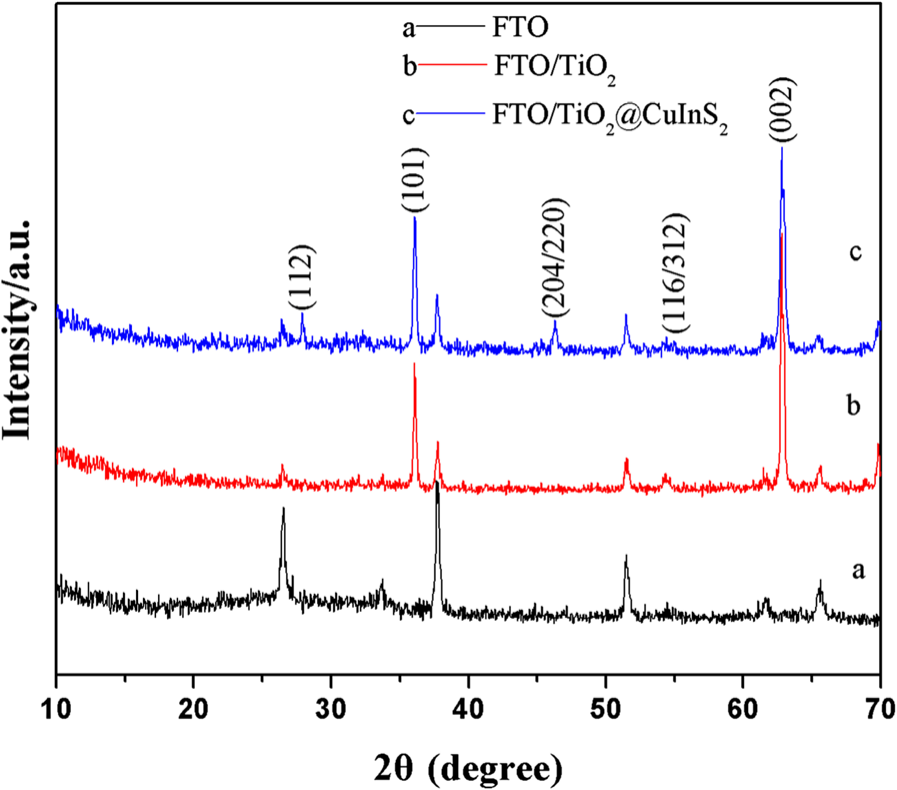
**XRD patterns.** (a) FTO substrate, (b) TiO_2_ NRA film grown on FTO substrate, and (c) CuInS_2_ absorbed TiO_2_ NRA film.

To further investigate the phase composition and phase structure of CuInS_2_ QD-modified TiO_2_ NRA films, XRD measurements were carried out. Figure 3 displays the X-ray diffraction patterns of the TiO_2_ NRA before and after modification with CuInS_2_. XRD shows that the TiO_2_ NRAs deposited on FTO substrate can be classified as tetragonal rutile. Eliminating the peaks originating from the FTO conductive glass (Figure 3a), all the diffraction peaks that appear upon nanorod growth films agree well with the tetragonal rutile phase (JCPDS file no.88-1175), which is in agreement with the HRTEM measurement. The significantly enhanced (002) peak in 2-theta of 63.20° indicates that the nanorods are well crystallized and grow perpendicular to the FTO substrate. As compared with curve (b), three additional peaks were observed after deposition with CuInS_2_ at 2*θ* = 27.9°, 46.5°, and 55.1° which can be indexed to the (112), (204)/(220), and (116)/(312) planes of tetragonal CuInS_2_, respectively (JCPDS No.85-1575). The mean diameter of CuInS_2_ particles was calculated to be approximately 8.27 nm by Scherrer equation, which is consistent with that observed in TEM image.

### Photoelectric properties of CuInS2 QD-sensitized TiO_2_ nanorod arrays

**Figure 4:**
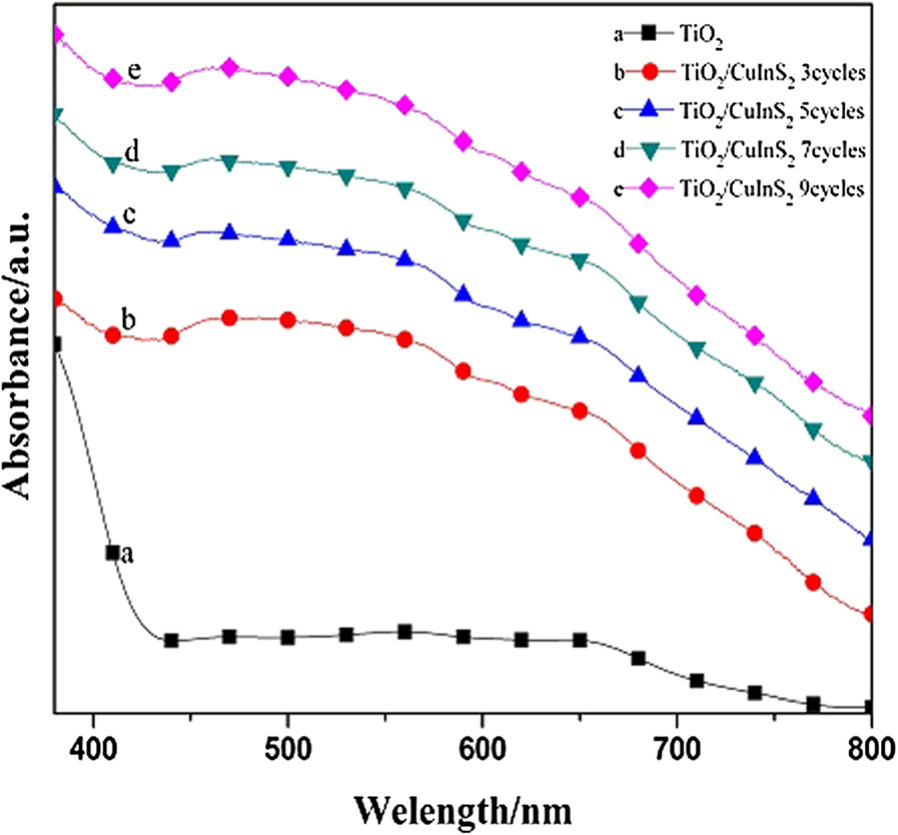
**Diffuse reflectance absorption spectra.** Bare TiO_2_ NRAs (curve a) and CuInS_2_ QD-coated TiO_2_ NRAs fabricated by the SILAR technique for 3 cycles (curve b), 5 cycles (curve c), 7 cycles (curve d), and 9 cycles (curve e), respectively.

The absorption spectra of bare TiO_2_ NRAs and CuInS_2_-sensitized TiO_2_ NRA electrodes fabricated with different SILAR cycles are compared in Figure 4. The TiO_2_ nanorod film exhibits an absorption edge in the ultraviolet region and has no significant absorbance for visible light because of its large energy gap (3.2 eV) [29]. For the TiO_2_ nanorod film sensitized with CuInS_2_, the light absorbance extends to the visible-light region, and the absorbance increases with increasing coating cycles, suggesting that the amount of CuInS_2_ deposited on TiO_2_ NRAs increased with the coating cycles. In addition to the increase of absorbance in the UV–vis spectra, the absorption edge undergoes a continual redshift with increasing coating cycles, indicating the growth of the CuInS_2_ QDs.

**Figure 5:**
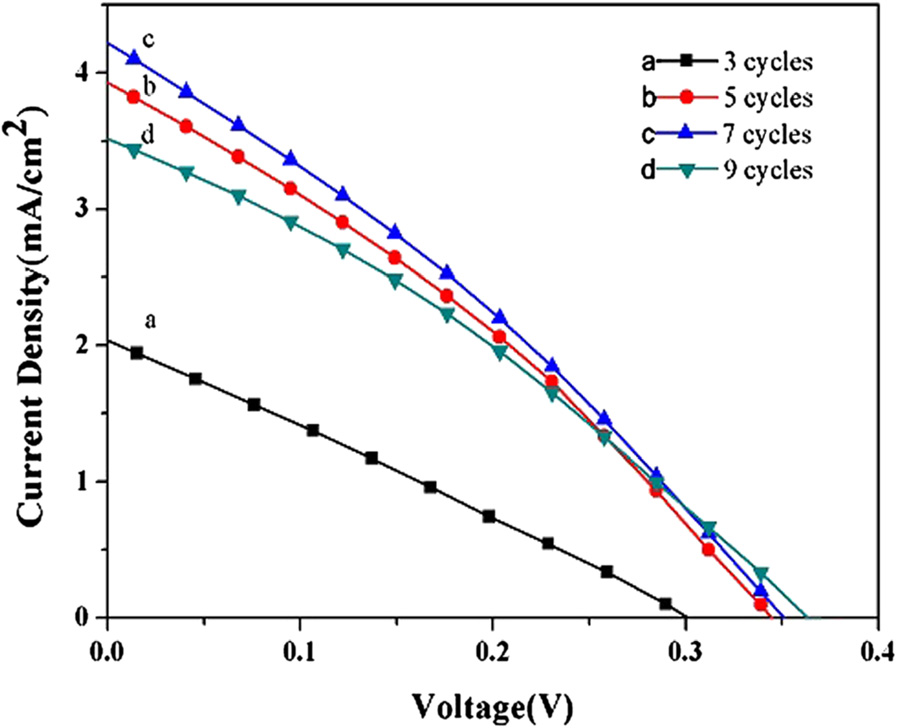
**Solar cell characterization of the devices with different CuInS_**2 **_coating cycles.** The curves were measured under AM 1.5 illumination with an active area of 0.16 cm^2^.

The CuInS_2_ QD-sensitized TiO_2_ NRA electrodes with different SILAR cycles of deposition were used as photoanodes in the sandwiched QDSSCs. The *I-V* curves measured under simulated AM 1.5 G sunlight illumination are shown in Figure 5. It is obvious from Figure 5 that the photovoltaic performance of the solar cells firstly increased in the initial 7 cycles, the optimum energy conversion efficiency was obtained after 7 cycles, with a short-circuit photocurrent of 4.22 mA cm^−2^, an open-circuit photovoltage of 0.36 V, a fill factor of 0.31, and an overall power conversion efficiency (*η*) of 0.46%, respectively. The enhancement of the photoelectrochemical properties can be illuminated as the result of increased light absorption in the visible light range, which has been indicated in Figure 4. With increasing SILAR cycles, the incorporated amount of CuInS_2_ on TiO_2_ NRAs gradually increased, which could not only contribute to absorb more photons to generate more photoexcited electrons, but also form a uniform and dense shell to reduce direct contact areas between the bare TiO_2_ surface and polysulfide electrolyte, consequently decreasing the probability of recombination from separated electrons in the TiO_2_ to the hole-transport material of electrolyte [30-32].

However, the *I*_sc_ and *η* were found to somewhat decrease when the coating cycles increased to 9 cycles. The possible reason for the reduced cell performance may be attributed to the aggregations and growth of the CuInS_2_ crystal nucleus, which will result in the presence of CuInS_2_ crystals with no direct contact with the TiO_2_, leading to higher recombination and thick sensitized layers blocking the infiltration of the electrolyte into the photoelectrode, thereby decreasing the regeneration efficiency of the photoelectrochemical cell [33,34]. Effects of SILAR cycles of CuInS_2_ on the photovoltaic performance of the QDSSCs are listed in Table 1. It is noteworthy that the best conversion efficiency of our cell is higher than the value of 0.38% obtained from presynthesized CuInS_2_ QDs directly attached to the TiO_2_ nanocrystalline films as the photoanode [17]. These results manifest the superiority of single-crystalline TiO_2_ NRAs to disordered TiO_2_ nanoparticle films when used as the host material in QDSSCs; also, it demonstrates that the SILAR process is a more preferable way for depositing semiconductor nanocrystalline sensitizers over TiO_2_ films.

**Table 1:** **Photovoltaic performance of the CuInS**_**2**_-**based QDSSC devices with different SILAR cycles**

**Photoelectrodes**	***J***_**sc**_	***V***_**oc**_**(V)**	**Fill factor (%)**	**Efficiency (*η*%)**
3 cycles	2.04	0.302	27	0.17
5 cycles	3.92	0.351	31	0.42
7 cycles	4.22	0.355	31	0.46
9 cycles	3.59	0.371	32	0.43

**Figure 6:**
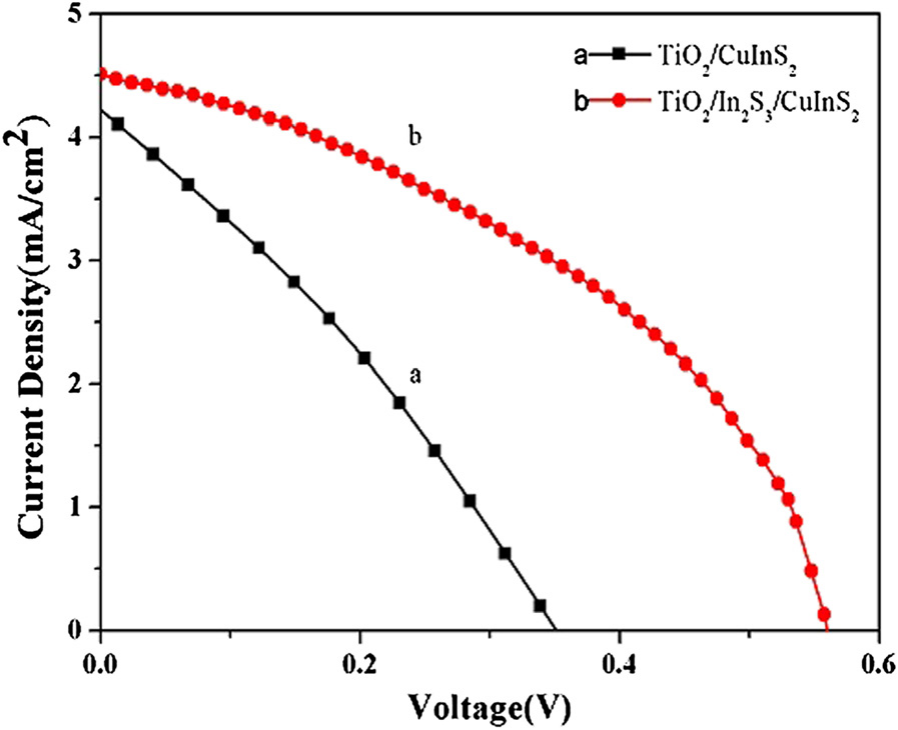
**J-V characteristics of different working electrodes measured under AM 1.5 global filter of 100 mW/cm**^**2 **^**sunlight.** Curve (a) TiO_2_/CuInS_2_(7) and curve (b) TiO_2_/In_2_S_3_(4)/CuInS_2_(7). The active surface area was 0.16 cm^2^.

In CuInS_2_-based thin-film solar cells, it has been revealed that there are unmatched band alignments and high surface state density existed in the heterostructure between TiO_2_ and CuInS_2_[17,35], which resulted in a high rate of recombination at the interface. Fortunately, this can be overcome by applying a buffer layer between TiO_2_ and CuInS_2_. Therefore, in order to modify the interfacial properties and to further improve the performance of CuInS_2_ QD-sensitized QDSSCs, a non-toxic In_2_S_3_ buffer layer also by SILAR was deposited on the TiO_2_ NRs before the deposition of CuInS_2_ (Additional file 2: Figure S3). The comparison of the photovoltaic performance and parameters of photoelectrodes with and without In_2_S_3_ buffer layer was shown in Figure 6 and Table 2, respectively. In the control experiment, *V*_oc_, FF, and then the efficiency increased dramatically in the presence of the In_2_S_3_ buffer layer, implying that the In_2_S_3_ buffer layer plays an important role in improving photovoltaic performance. Additional file 3: Figure S4 demonstrates the dark current–voltage characteristic curves of CuInS_2_-based QDSSC with and without In_2_S_3_ buffer layer. The dark current results from the reduction of electrolyte by the conduction band electrons of TiO_2_. The onset of the dark current of QDSSC with In_2_S_3_ buffer layer occurs at the higher forward bias, which indicates that the dark reaction was efficiently suppressed by applying a buffer layer of In_2_S_3_. This can be explained by the forming of cascade band structures at the TiO_2_/In_2_S_3_/CuInS_2_ interface (Additional file 4: Figure S6), which suppresses the back flow of electrons and restrains the electron–hole recombination [35-37].

**Table 2:** Photovoltaic performance for CuInS_**2**_-sensitized TiO_**2 **_NRA photoelectrodes with and without In_**2**_**S**_**3 **_buffer layer

**Photoelectrodes**	***J***_***sc***_***I *(mA/cm**^**2**^**)**	***V***_**oc**_**(V)**	**Fill factor (%)**	**Efficiency (*η*%)**
TiO_2_/CuInS_2_(7)	4.22	0.355	31	0.46
TiO_2_/In_2_S_3_(4)/CuInS_2_(7)	4.51	0.559	41	1.06

It should be mentioned that the efficiency of the CuInS_2_-based QDSSCs present in our study is still limited, which may be attributed to the limitation of TiO_2_ NR length. The typical thickness of TiO_2_ nanoparticle films is about 13 μm, but for TiO_2_ NR array films used in our experiment, the length was just about 3 μm. As a result, although there are many advantages of 1D TiO_2_ NRs, the insufficient length resulted in poor QD loadings and light harvesting, which constrained the efficiency of TiO_2_ NR cells to relatively lower levels than that of nanoparticle-based ones. To further improve photovoltaic performances of 1D nanostructure-based QDSSCs, it is necessary to pay more attention to the internal surface area of TiO_2_ NRAs and interfacial properties that are very critical to determine the fate of excitons generated inside the semiconductor QDs.

## Conclusions

In this study, for the first time, we have employed a facile SILAR process to deposit CuInS_2_ QD onto TiO_2_ NRAs, which was prepared by a simple hydrothermal method. The CuInS_2_ QD-sensitized TiO_2_ NRAs were used as photoanodes to assemble sandwiched QDSSCs. The effect of SILAR cycles on the photoelectrochemical performance of the CuInS_2_-sensitized solar cells was investigated. With optimal CuInS_2_ SILAR cycles and introduction of In_2_S_3_ buffer layer to modify the interface, the best photovoltaic performance with an energy conversion efficiency of 1.06% under AM 1.5 G illuminations, an open-circuit photovoltage of 0.56 V, a short circuit current density of 4.51 mA cm^−2^, and a FF of 0.41 were achieved. The present CuInS_2_-based QDSSC fabrication approach combined the advantages of 1D TiO_2_ NRAs and *in situ* growing of the target semiconductor sensitized layers and buffer layer by SILAR, which can be used for construction of other useful optoelectronic devices and composite catalysts.

## Competing interests

The authors declare that they have no competing interests.

## Authors’ contributions

ZZ is the primary author and participated in the experiment design, experiment analysis, interpretation of data, and language modification. SY and JF carried out the experiments, characterization, and acquisition of data. ZH and WZ participated in the discussion. ZD and SW are the investigators who helped in the analysis and interpretation of data, drafting of the manuscript, and making revisions. All authors read and approved the final manuscript.

## Authors’ information

ZZ is a Ph.D. candidate in the Key Laboratory for Special Functional Materials of Ministry of Education, Henan University. SY, JF, and ZH are all masters degree students on Inorganic Material Chemistry. WZ is a Ph.D. degree holder on Analytical Chemistry. ZD is the distinguished professor and research director in the Key Laboratory for Special Functional Materials of Ministry of Education. SW is a full professor on Material Chemistry and Physics.

## Supplementary Material

Additional file 1 **Figure S1.** EDS spectrum of the CuInS_2_ QD-sensitized TiO_2_ NRA photoelectrode after annealed in sulfur ambiance at 500°C for 30 min. The ratio of Cu/In/S is 1.02:1.00:1.91. Click here for file

Additional file 2 ** Figure S2.** SAED patterns of TiO_2_ NR (a) and CuInS_2_ QDs (b).Figure S3. EDS spectra of TiO_2_ NRA photoelectrode after In_2_S_3_ deposition. Click here for file

Additional file 3 ** Figure S4.** Dark current–voltage characteristic curves of CuInS_2_-based QDSSC with (red dots) and without (black squares) In_2_S_3_ buffer. Figure S5. IPCE spectra of CuInS_2_ QD-sensitized solar cell with different SILAR cycles. Click here for file

Additional file 4 ** Figure S6.** Band diagram of CuInS_2_ QD-sensitized solar cell. Buffer layers of In_2_S_3_ are applied to suppress electron–hole recombination at the interface. Click here for file
