# Supplementary material for: CuInS2 quantum dot-sensitized TiO2 nanorod array photoelectrodes: synthesis and performance optimization
Source: Nanoscale Res Lett. 2012 Nov 27;7(1):652. doi: 10.1186/1556-276X-7-652 (PMC3552836; doi:10.1186/1556-276X-7-652)
Supplement: Additional file 1 — Figure S1. EDS spectrum of the CuInS2 QD-sensitized TiO2 NRA photoelectrode after annealed in sulfur ambiance at 500°C for 30 min. The ratio of Cu/In/S is 1.02:1.00:1.91. [file 1556-276X-7-652-S1.doc]

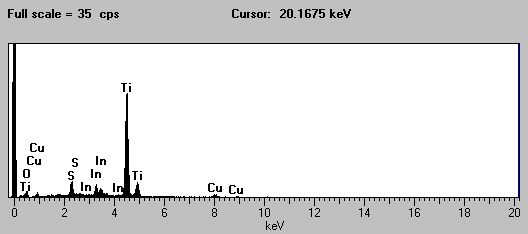
**Figure S1.** EDS spectrum of the CuInS_2_ QD-sensitized TiO_2_ NRA photoelectrode after annealed in sulfur ambiance at 500 ℃ for 30 min. The ratio of Cu:In:S is 1.02:1.00:1.91.
