# Supplementary material for: CuInS2 quantum dot-sensitized TiO2 nanorod array photoelectrodes: synthesis and performance optimization
Source: Nanoscale Res Lett. 2012 Nov 27;7(1):652. doi: 10.1186/1556-276X-7-652 (PMC3552836; doi:10.1186/1556-276X-7-652)
Supplement: Additional file 2 — Figure S2. SAED patterns of TiO2 NR (a) and CuInS2 QDs (b).Figure S3. EDS spectra of TiO2 NRA photoelectrode after In2S3 deposition. [file 1556-276X-7-652-S2.doc]

(a)

(b)

**FigureS2.** SAED patterns of TiO_2_ NR (a) and CuInS_2_ QDs (b).

**FigureS3.** EDS spectra of TiO_2_ NRA photoelectrode after In_2_S_3_ deposition.
