# Supplementary material for: CuInS2 quantum dot-sensitized TiO2 nanorod array photoelectrodes: synthesis and performance optimization
Source: Nanoscale Res Lett. 2012 Nov 27;7(1):652. doi: 10.1186/1556-276X-7-652 (PMC3552836; doi:10.1186/1556-276X-7-652)
Supplement: Additional file 3 — Figure S4. Dark current–voltage characteristic curves of CuInS2-based QDSSC with (red dots) and without (black squares) In2S3 buffer. Figure S5. IPCE spectra of CuInS2 QD-sensitized solar cell with different SILAR cycles. [file 1556-276X-7-652-S3.doc]

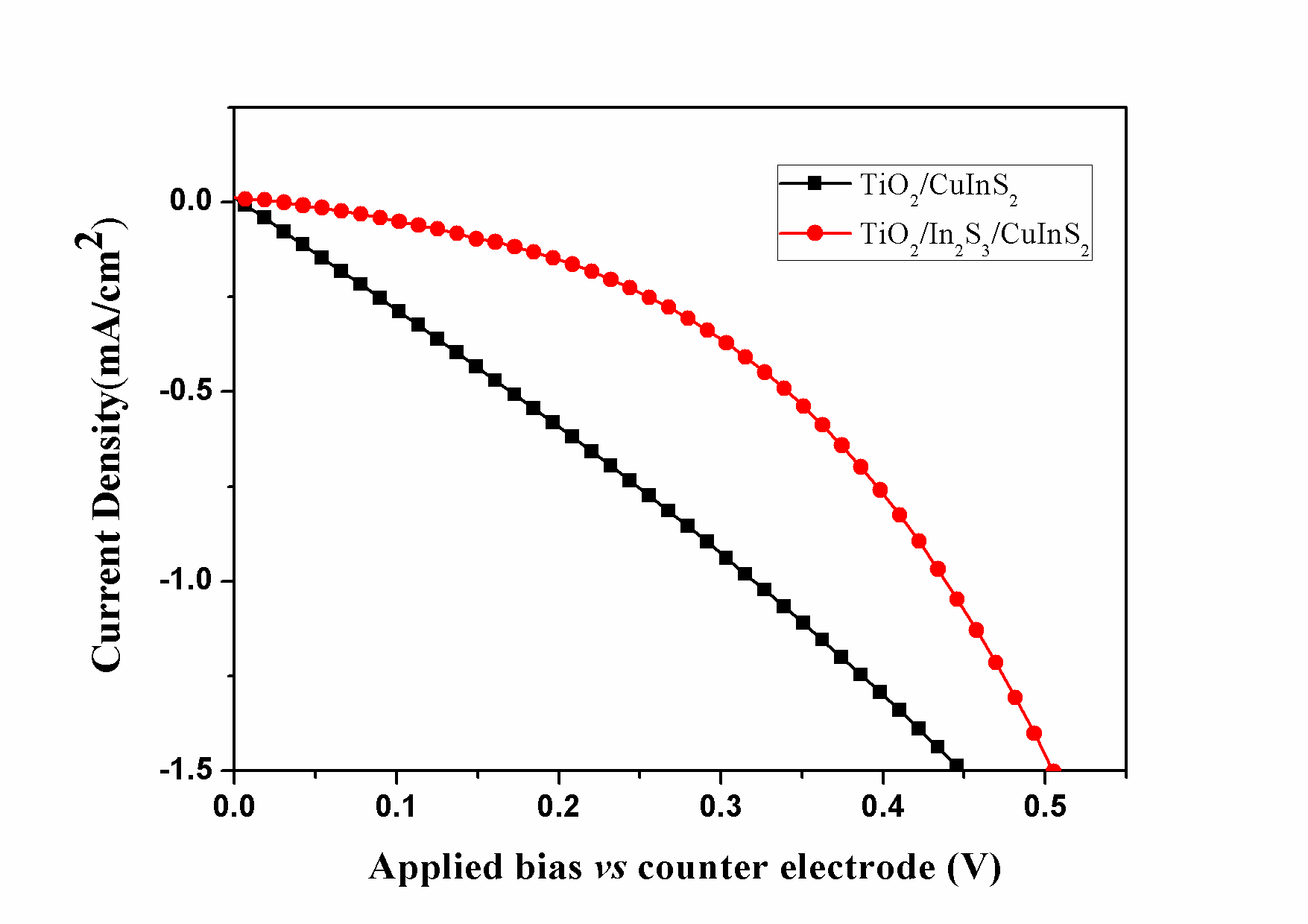


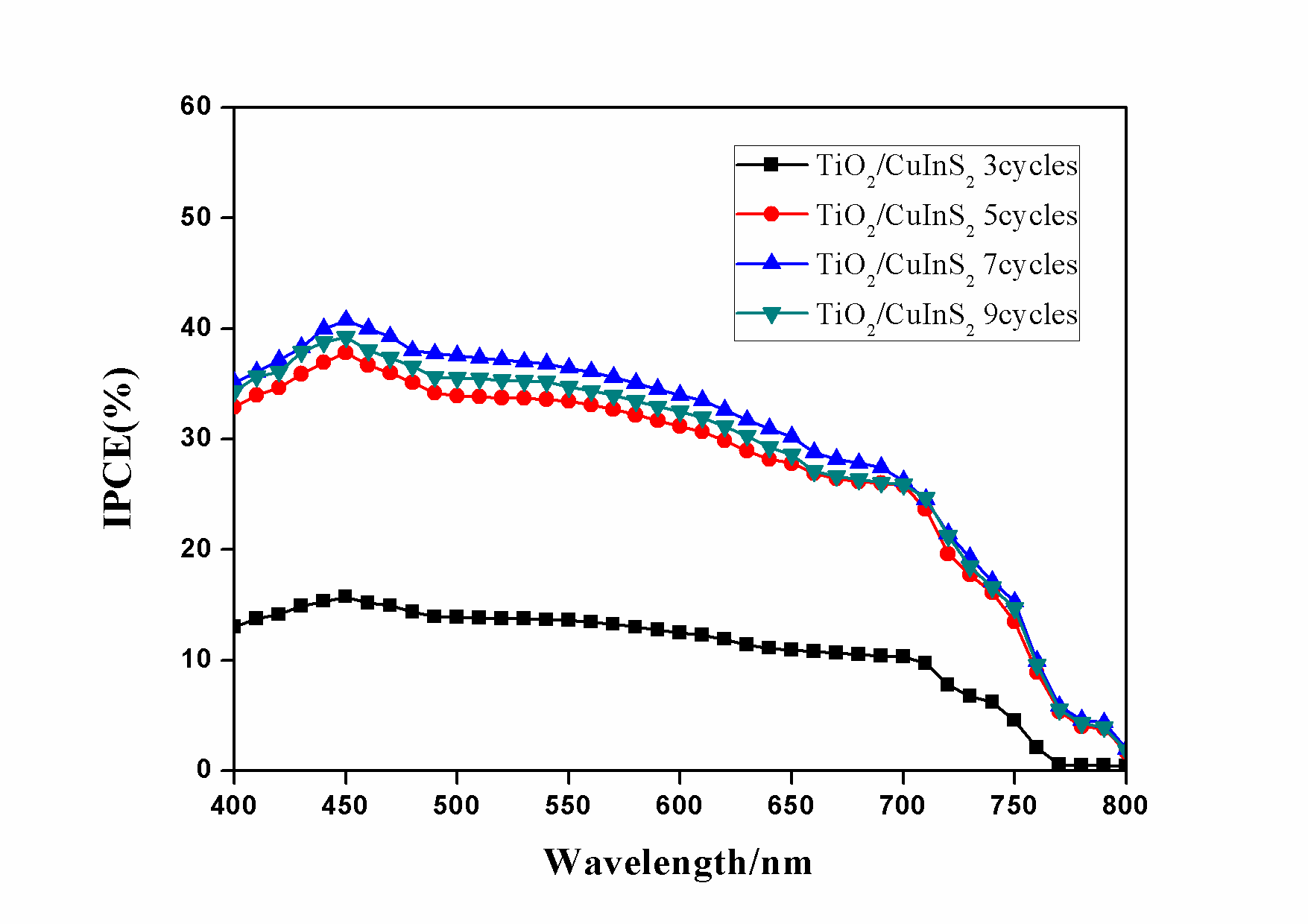
**FigureS4.** Dark current-voltage characteristic curves of CuInS_2_ based QDSSC with (red dots) and without (black squares) In_2_S_3_ buffer layer.

**FigureS5.** IPCE spectra of CuInS_2_ QD-sensitized solar cell with different SILAR cycles.
