# Supplementary material for: CuInS2 quantum dot-sensitized TiO2 nanorod array photoelectrodes: synthesis and performance optimization
Source: Nanoscale Res Lett. 2012 Nov 27;7(1):652. doi: 10.1186/1556-276X-7-652 (PMC3552836; doi:10.1186/1556-276X-7-652)
Supplement: Additional file 4 — Figure S6. Band diagram of CuInS2 QD-sensitized solar cell. Buffer layers of In2S3 are applied to suppress electron–hole recombination at the interface. [file 1556-276X-7-652-S4.doc]

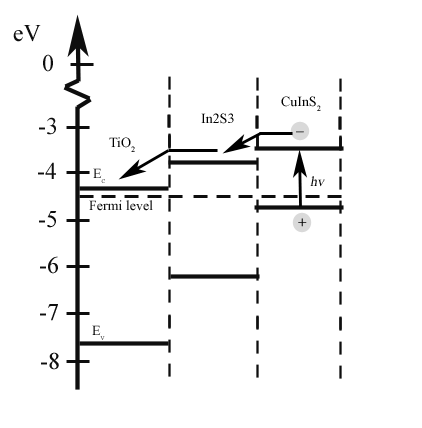


**FigureS6.** Band diagram of CuInS_2_ QD-sensitized solar cell. Buffer layers of In_2_S_3_ are applied to suppress electron–hole recombination at the interface.
